# Amino Acid Profile and Biological Properties of Silk Cocoon as Affected by Water and Enzyme Extraction

**DOI:** 10.3390/molecules26113455

**Published:** 2021-06-07

**Authors:** Chuleeporn Bungthong, Colin Wrigley, Thanathat Sonteera, Sirithon Siriamornpun

**Affiliations:** 1 Research Unit of Process and Product Development of Functional Foods, Department of Food Technology and Nutrition, Faculty of Technology, Mahasarakham University, Kantarawichai, Mahasarakham 44150, Thailand; chuleeporn.bt@bru.ac.th; 2 Queensland Alliance for Agriculture and Food Innovation (QAAFI), University of Queensland, Brisbane, QLD 4072, Australia; c.wrigley1@uq.edu.au; 3 Managing Director, Siam Natural Products Co., Ltd., Sutthisan Winitchai Road, Samsen Nai, Khet Phaya Thai, Bangkok 10400, Thailand; ole.sonteera@gmail.com

**Keywords:** silk protein, protease, serine, glutamic acid, α-amylase, bitter taste

## Abstract

We compared the efficacy for protein extraction of water versus enzymatic extraction. The amino-acid composition, inhibitory activity against enzymes α-amylase and α-glucosidase, and anti-glycation activities of silk protein extract (SPE) were determined. We used water extraction (100 °C, six hours) and protease-enzymatic extraction. The microstructure of silk fibers was obviously different after extraction. The results showed that enzymatic extraction gave the greater values of protein content, amino acids, total phenolic content (TPC), and total flavonoid content (TFC), as well as all biological activities parameters tested, but it also provided a more bitter taste in the extract—contributing amino acids of 51% (arginine, phenylalanine, histidine, valine, tryptophan, isoleucine, and leucine) and less sweet and umami taste contributing amino acids than did water extraction, which could be more suitable to be used as concentrated nutraceuticals.

## 1. Introduction

Silk is a protein fiber that consists of sericin and fibroin; of these, fibroin protein comprises 70% to 80% of the silk fiber. It has amorphous and crystalline domains with short amino-acid chains that allow it to maintain its compact structure [1]. Sericin is a globular protein that constitutes about 20–30% of the silk fiber. Its role is to coat and link the fibroin filaments together in the silkworm cocoons [2]. The proteins, sericin and fibroin, have been widely studied for medical applications due to their good physiochemical properties and biological activities, such as anti-oxidation, anti-inflammatory, biocompatibility, acceleration of cell proliferation, and activation of collagen synthesis. In the silk industry, sericin is removed from the fibroin and subsequently discarded. However, it has been shown that this protein presents interesting properties, which may allow its application in several fields. Due to its moisturizing and anti-wrinkling abilities, sericin is an interesting compound to the cosmetic industry [3,4]. Additionally, sericin has been studied for its biomedical purposes, such as a biomaterial and for drug delivery [5,6,7,8], mainly because it has been reported to be immunologically inert [6]. Sericin’s antioxidant activity is one of its most significant properties because it could provide positive effects for human health and in the food industry as a natural food preservative [9].

Our previous study showed that silk cocoons, extracted with boiling water for six hours, provided the best results with respect to amino-acid profiles, total phenolic and flavonoid contents, and biological properties, including anti-glycation, α-amylase, and α-glucosidase inhibition [10]. However, although our results revealed that longer extraction time offered greater biological values, more concentrated extract is required for making nutraceuticals or cosmetics besides functional foods. To explore more potential uses of silk cocoon components, other extraction methods need to be employed, such as (1) extraction with detergents and soaps that lead to protein denaturalization and partial hydrolysis of the silk filament chains [5]: sodium carbonate [11], calcium hydroxide [12], and non-ionic detergents [13] have been utilized for the degumming process, (2) extraction with steam using autoclave: due to the high temperature applied, hydrogen bonds between hydroxyl groups become unstable allowing the water to interact with polar amino acids of the sericin [14]. The molecular weight of the samples obtained is in the range of 27 to 200 kDa [15], (3) enzyme-aided extraction, etc. Enzyme extraction is normally used for fractionation of plant raw material and for extraction of molecules in a harmless manner [16].

There have been many studies using enzymic extraction for quality improvement. For instance, Sangwong et al. [17] used a protease enzyme to modify sericin extracted from cocoons; the results obtained in this study suggest that sericin protein, modified using a protease enzyme, could be adopted as an ingredient in food and cosmetic applications. The enzyme dosage and treatment time influence the kinetics of the process. Moreover, chemical properties of soluble sericin peptides vary according to the enzyme utilized. Peptides in the range of 5 to 20 kDa are obtained and these are free of alkali and fatty acids [18]. Whereas this approach is slightly more expensive than the techniques described above, less energy is required. Consequently, this process becomes more sustainable [19]. The combined use of enzymes (*savinase* and *alcalase*) and ultrasound to extract the sericin from the silk fiber was also assessed. In this case, the efficacy of the degumming process increased along with the treatment time. However, the integrity of the sericin isolated was not studied [13]. Similarly, a thermostable alkaline serine protease from a bacterium (*Bacillus hallodurans*) capable of removing sericin from silk was recently isolated. This novel protease has exhibited a higher degumming ability than commercial Alcalase proteases [20]. However, there is little information reported on the comparison of the chemical composition and biological properties of Thai silk cocoons from water and enzymatic extraction. 

Therefore, we aimed to compare water versus enzymatic extraction on amino-acid profiles, total phenolic and flavonoids contents, biological properties, anti-glycation, α-amylase, and α-glucosidase inhibition with respect to optimizing the extraction method of silk proteins. The Nangtui strain was selected as the most popularly cultivated in North-eastern Thailand (*Bombyx mori*; yellow cocoon). We expected to generate useful information for wider uses of this silk protein extract. This study should also provide a useful foundation for improving the processing and production of functional food products.

## 2. Results and Discussion

### 2.1. Microstructure of Silk Fibers

**Figure 1:**
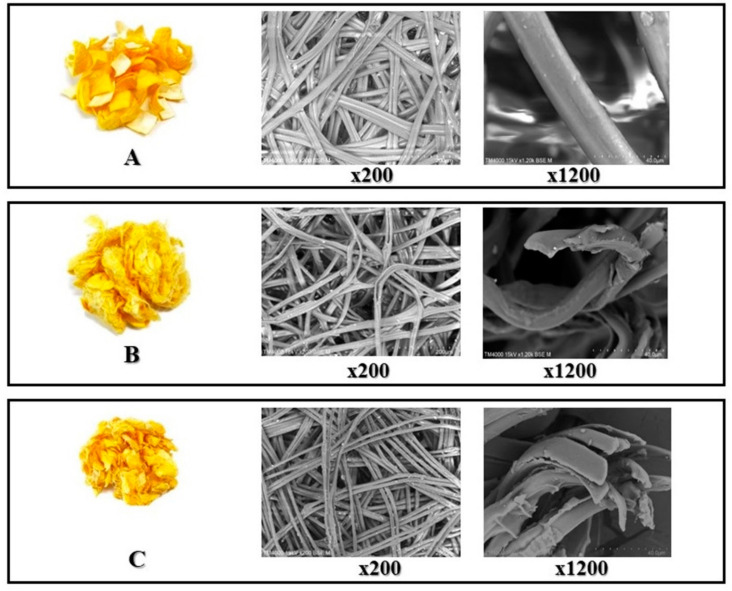
Appearance and microstructure of silk fiber. (**A**) Control treatment that was immersed in water only and without heating, (**B**) water extraction and (**C**) enzymatic extraction, using scanning electron micrographs (SEM; ×200 and ×1200).

The microstructure of silk fibers was obviously different after extraction. SEM and expanded image sizes at 200× and 1200× showed that the resulting silk fibers differed comparing water and enzymatic extraction methods. It is possible to observe that the control has a large and smooth surface when compared to water extraction, which produced a silk thread that was shrunken, with some fracture, when compared with enzymatic extraction. This latter method produced threads that were very small and shrunken (Figure 1). These results were in agreement with Wongpinyochit et al. [21], who speculate that proteolytic enzymes disturbed the silk packing geometry, permitting greater water ingress, resulting in particle swelling. Simultaneously, degradation reduced surface charges, enabling degradation products to loosely accumulate on the remaining particle surface.

### 2.2. The Difference Due to Extraction Method on the Amino-Acid Content of Silk Protein Extract (SPE) and Protein Content

The extraction of SPE with enzymes provides a total essential amino-acid value that is three times higher than water-extraction methods (Table 1), due to the use of a protease enzyme which broke the peptide bonds of the protein molecules producing free amino acids and shorter peptides. Protein degradation has the advantage that enzymes are highly specific to the respective substrate. Therefore, large amounts of enzymes are not needed, and they can degrade proteins in mild conditions. In addition, the use of enzymes has a relatively high protein-degradation rate when compared with the use of acids or alkalis. Enzymes are biological catalysts which accelerate the rates of a wide variety of chemical reactions. Different enzymes may cause hydrolysis, reduction, oxidation, coagulation and decomposition reactions. Proteolytic enzymes or proteases are those enzymes which hydrolytically cleave the peptide bond that links amino acids together in the polypeptide chains, thus degrading the proteins into smaller molecules, such as peptones, peptides, and amino acids. These products of biotechnological processes are finding wider applications in medicine and industry; the use of various proteolytic enzymes has, therefore, increased enormously in recent years [22,23,24]. The result of amino acid resulted in enzymatic extraction has a higher protein content, as well. The extraction by enzyme gives the value protein content of 3 mg/g (Table 1). The extraction yield of SPE in the water and hydrolysate using enzyme were 43.31 and 94.61%, respectively. The extraction of cocoons by water takes a long time, causing a lot of water to evaporate resulting in less yield. In contrast to the use of enzymes, higher yields was obtained.

### 2.3. The Effect of Extraction Method on Amino-Acid Contributions to Taste

**Figure 2:**
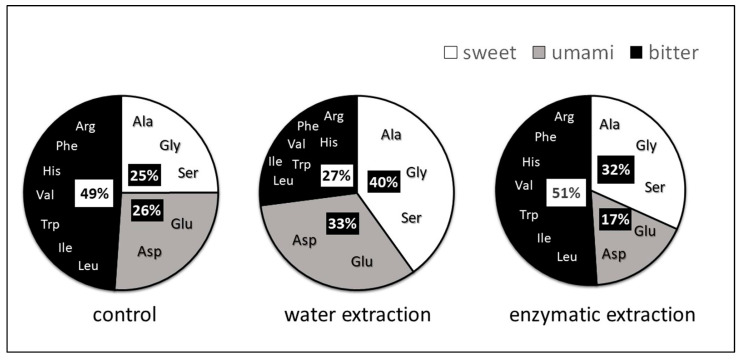
The effect of extraction method on the contribution of amino acids to the taste of SPE.

The SPE obtained by extraction with either water or enzyme, affected the contributions to taste (alanine, glycine and serine are sweet tasting, arginine, phenylalanine, histidine, valine, tryptophan, isoleucine, and leucine have bitter taste, and glutamic acid and aspartic acid are umami). The resulting taste differences (1; sweet, 2; umami: is the fifth basic taste, in addition to sour, sweet, salty, and bitter taste that produces delicious taste, and 3; bitter) are shown in Figure 2. Extraction with water or enzyme gives the values of sweet, umami and bitter taste of 40, 33, 27% and 32, 17, 51%, respectively. However, enzymatic hydrolysis of proteins may produce compounds with a more bitter taste than water extraction due to the enzymic liberation of amino-acid hydrophobic groups, such as isoleucine, phenylalanine, tryptophan, tyrosine, and valine [25]. Proteases with a broad specificity have a tendency of hydrolyzing at hydrophobic amino-acid residues, leaving a non-polar amino-acid residue at the C-terminus of the peptide formed. This leads to relatively greater bitterness [26].

### 2.4. The Effect of Extraction Method on Total Phenolic Content and Total Flavonoid Content

**Figure 3:**
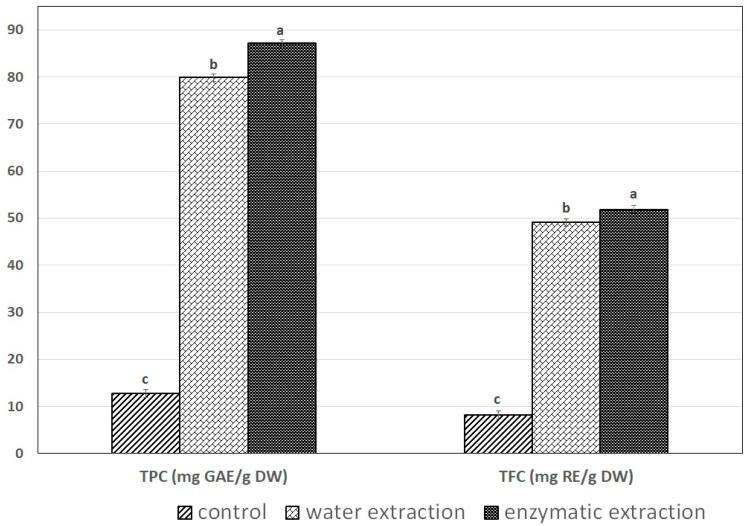
The effect of extraction method on total phenolic (TPC) and total flavonoid (TFC) contents of SPE. Values are expressed as mean ± standard deviation (*n* = 3). Means with different letters on different bars were significantly different among treatments at *p* < 0.05.

The enzymatic extraction of SPE showed the highest total phenolic content (TPC) and total flavonoid content (TFC) of 87.65 mg GAE/g DW and 52.02 mg RE/g DW, respectively (Figure 3), when compared with water extraction (with significant differences among the method (*p* < 0.05). The phenolic and flavonoid profiles were also studied. The main phenolic acids found were gallic acid, protocatechuic acid, p-hydroxybenzoic acid, ferulic acid and sinapic acid, whereas the major flavonoids found were rutin and myricetin (data not shown). Protease treatment of SPE significantly increased polyphenol and flavonoid contents. Many reports have reported that the physiological function of natural foods can be attributed to the antioxidative capacity of their antioxidant components. The protease may have acted by catalyzing the hydrolytic degradation of intracellular organelles and cell membranes of protein nature [27,28,29]. The increased release of polyphenols for the enzyme preparations suggests that these enzymes may contain activities that directly promote selective release of antioxidant phenols or modify released phenols to become more potent antioxidant compounds. Aside from the favorable effects of enzyme-aided extraction on bioactive compounds, a decrease in the anthocyanin content has been reported in fruit juice [30].

### 2.5. The Effects of Exteaction Method on DPPH Radical-Scavenging Activity, ABTS^+•^ and the FRAP Assay

**Figure 4:**
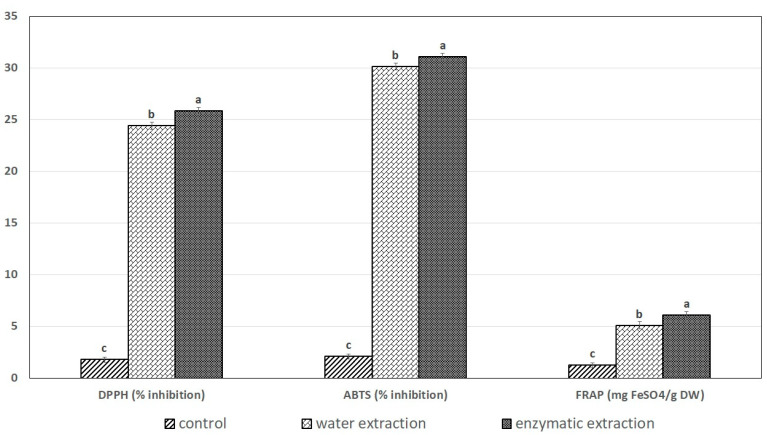
The effect of extraction method on DPPH radical-scavenging activity, ABTS^+•^ assay, and FRAP assay of SPE. Values are expressed as mean ± standard deviation (*n* = 3). Means with different letters on different bars were significantly different among treatments at *p* < 0.05.

The antioxidant activity of SPE with different extraction methods was evaluated by determining DPPH radicals, ABTS^+•^, and FRAP Assay, as shown in Figure 4. The free radical-scavenging activities of SPE with different extraction methods are indicated by % inhibition. SPE from enzymatic extraction showed the highest levels of DPPH radicals, ABTS^+•^, and FRAP of 26.32, 32.08, and 6.24% inhibition, respectively, while water extraction showed the lowest of 24.85, 30.19, and 5.01% inhibition, respectively (with significant differences among the method (*p* < 0.05). There are many reports that the extraction method induces conformational and functional changes in proteins, including antioxidant activity [17,30]. Enzyme-aided extraction has been reported to have both adverse and favorable effects on bioactive compounds. Increases in antioxidant activity by enzyme-aided extraction have been reported by many studies, mostly in plants [31]. For instance, Wanyo et al. [32] reported that cellulase-treated ground rice husk could improve its biological activities, thus providing more acceptable product. The increase or decrease in ferric-reducing power for protein hydrolysates may be related to the exposure of electron-dense amino-acid side-chain groups, such as polar or charged moieties during hydrolysis [33].

### 2.6. Inhibitory Activity against the α-Amylase, α-Glucosidase and Anti-AGEs Formation Activity in SPE

**Figure 5:**
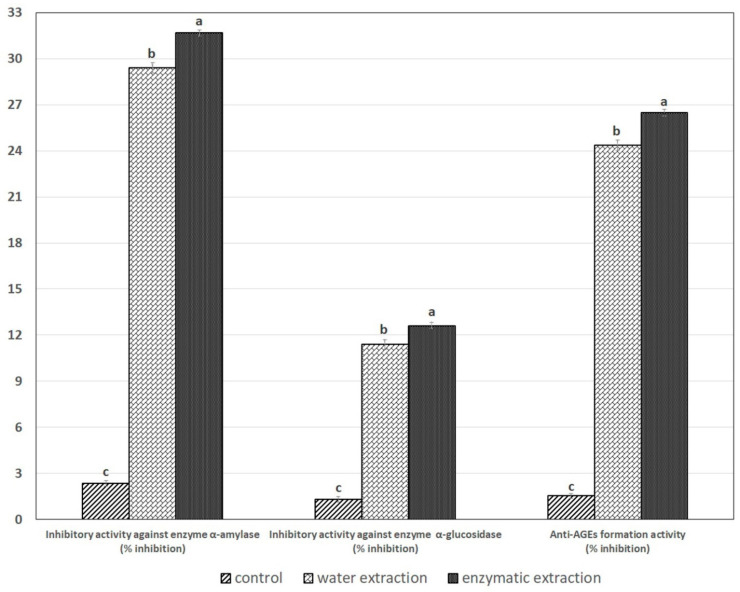
The effect of extraction method on inhibitory activity against enzyme α-amylase and α-glucosidase and antiglycation activities of SPE. Values are expressed as mean ± standard deviation (*n* = 3). Means with different letters on different bars were significantly different among treatments at *p* < 0.05.

The SPE had inhibitory activity against the enzymes α-amylase and α-glucosidase. This activity was highest for enzymatic extraction, which provided better inhibition than water-extracted protein (Figure 5). Inhibitory activity against the α-amylase and α-glucosidase of SPE from water and enzymatic extraction were 29.18 and 11.23% inhibition, respectively, and the enzymatic extractions were 31.86 and 12.69% inhibition, respectively (with significant differences among the method (*p* < 0.05). Some researchers have reported that several amino acids act as α-glucosidase inhibitors. Suzuki et al. [34] had initially tested the inhibitory effects of Gly or Ser and showed some α-glucosidase inhibition activity. Various in vitro assays have shown that many plant polyphenols possess carbohydrate-hydrolyzing enzyme inhibitory activities. Some compounds, such as green tea polyphenols, sweet potato anthocyanins, the soy isoflavone genistein, and several flavonoids, inhibit α-glucosidase and α-amylase activities [35,36]. These inhibitory activities of plant phytochemicals against carbohydrate-hydrolyzing enzymes contribute to lowering postprandial hyperglycemia. α-Glucosidase inhibitors, such as acarbose, competitively bind to the oligosaccharide binding site of α-glucosidase or α-amylase, which prevents the binding and enzymatic hydrolysis of the oligosaccharide substrate [37]. It is, therefore, possible that the α-glucosidase inhibitory activities of these compounds result from the phenol group of acarbose and polyphenols binding to these enzymes.

That the SPE have the ability of anti-AGEs formation activity can be found in both extraction methods (water and enzymatic extraction), with 24.33 and 26.83% inhibition, respectively (with significant differences among the method (*p* < 0.05). However, enzymatic extraction provided better inhibition than water extraction (Figure 4). The anti-glycation capacity of numerous medicinal herbs and dietary plants was comparable, or even stronger than that of aminoguanidine [38]. Several studies have demonstrated that the anti-glycation activity correlates significantly with the phenolic content of tested plant extracts [39,40]. Polyphenols are the most abundant dietary antioxidants, being common constituents of fruits, vegetables, cereals, seeds, nuts, chocolate, and beverages, such as coffee, tea, and wine. They have been shown to lead to many health benefits, such as prevention of cancer [41], neurodegenerative diseases [42], cardiovascular diseases, and diabetes [43]. Therefore, SPE (water or enzyme extracts) with total flavonoid contents of 49.64 and 52.02 mg RE/g DW, respectively, thus, would be capable of causing inhibitory activity against the enzymes α-amylase, α-glucosidase, and anti-AGEs formation activity.

Several investigations have found a significant correlation between protein hydrolysate antioxidant activity and specific amino acid residues [44]. For example, Zainol et al. [45] found that aromatic amino acids (such as tyrosine and phenylalanine) can help to scavenge free radicals by functioning as potent electron donors, while hydrophobic amino acids (such as alanine, leucine, and proline) can also aid to scavenge free radicals. Peptides rich in methionine, leucine, histidine, alanine, and valine have a high antioxidant activity [46]. As a result of the high quantity of these amino acid residues with enhanced antioxidant activity in the hydrolysate generated by alcalase, the hydrolysate should have excellent antioxidant potential.

## 3. Materials and Methods

### 3.1. Silk Cocoons

Yellow Thai-silk cocoons (Nangtui strain) came from Thailand’s Phutthaisong District, in the Buriram Province.

### 3.2. Chemicals and Reagents

Essential and non-essential amino-acid standards came from Sigma–Aldrich Co. (St. Louis, MO, USA). Sigma–Aldrich provided the Folin–Ciocalteu reagent, 2,2-diphenyl-1-picrylhydrazyl (DPPH),2,4,6-tripiridyl-s-triazine (TPTZ), 2,2′-azino-bis (3-thylbenzthiazoline- 6-sulphonic acid) (ABTS), α-amylase (from Aspergillus oryzae), α -Glucosidase (from Bacillus stearothermophilus), *p*-nitrophenyl—d-glucopyranoside (PNP-G), bovine serum al-bumin (BSA), sodium azide, and protease (from Bacillus licheniformis). Reagents for the HPLC came from Merck, Darmstadt, Germany.

### 3.3. Extraction Methods

(1) Control treatment was that of Sangwong et al. [17]. Small pieces of silk cocoon (two grams) were immersed in 200 mL distilled water for two hours (without heating) and centrifugated for ten minutes at 5000× *g* (Universal 320/320R, Hettich, Boucherville, QC, Canada). The extracts were filtered through Whatman No. 1 paper under vacuum to remove insoluble material. Silk protein extract (SPE) was the final product, kept at −18 °C in an amber bottle.

(2) The water-extraction method was that of Bungthong and Siriamornpun [10]. Small pieces of silk cocoon (two grams) were extracted at 100 °C for 6 h in 200 mL distilled water. The centrifugation, filtration, storage process of SPE was performed in the same way as for control treatment.

(3) Enzyme treatment was that of Vaithanomsat and Punyasawon [47]. Small cocoon pieces soaked in distilled water over night at 10 °C were treated (cocoon:water ratio of 1:100 with the Alcalase^®^ from Novozymes 0.5% (*v/v*) (pH adjusted to 8.0) and incubated (50 °C) for 120 min. To stop enzyme action, the mixture was heated to 100°C for twenty minutes and then centrifuged (6000× *g* for 15 min). The filtration and storage process of SPE was performed in the same way as for control treatment.

### 3.4. Microstructure of Silk Fibers

A light microscope (Carl Zeiss Inc., Toronto, ON, Canada) was used to examine section slides of silk fibers [48]. Zeiss xioCamlCc3 program was used to digitize the files. A scanning electron microscope (SEM; TM4000 plus, Hitachi, Japan) fitted with the TM 4000 plus software was used to examine structural improvements. Prior to SEM analysis in vacuum mode, samples were coated with gold.

### 3.5. Protein Determination

To determine protein content, we used the Bradford assay [49]. SPE (0.5 mL) plus Bradford solution (1.0 mL) was incubated for 5 min. Bovine serum albumin (BSA) was used as standard. Absorbance was determined at 595 nm with a visible spectrophotometer (DR 2700™ Portable Spectrophotometer, Hach, Loveland, CO, USA).

### 3.6. Amino-Acid Content by LCMS/MS

Amino-acid content was determined according to Chumroenphat et al. [50]. We used an LC–MS–MS (LCMS-8030, Shimadzu, Kyoto, Japan) triple-quadrupole mass spectrometer in electrospray ionization (ESI) mode.

### 3.7. Total Phenolic Content (TPC)

The total phenolic content was determined according to Kubola and Siriamornpun [51]. We used a spectrophotometer (UV-1700, Shimadzu, Tokyo, Japan) to measure the absorbance of the solution samples at 725 nm. TPC was measured in milligrams of gallic acid equivalents (GAE) per gram of dry weight (mg GAE/g DW).

### 3.8. Total Flavonoid Content (TFC)

The total flavonoid content was determined according to Kubola and Siriamornpun [51]. A spectrophotometer (UV-1700, Shimadzu, Tokyo, Japan) was used to calculate the absorbance at 510 nm. The results were expressed in milligrams of rutin equivalents (RE) per gram of dry weight (mg RE/g DW).

### 3.9. DPPH Radical Scavenging Activity

DPPH radical scavenging activity was determined according to Brand-Williams et al. [52]. A spectrophotometer (UV-1700, Shimadzu, Tokyo, Japan) was used to determine the absorbance at 517 nm. The DPPH inhibition was calculated using the following equation:

Inhibition (%) = [(Abs. control − Abs. sample)/Abs. control] × 100,(1)

where the Abs. sample = absorbance of sample, and Abs. control = absorbance of the control.

### 3.10. Antioxidant Activity by ABTS Assay

To evaluate the free radical-scavenging results, the ABTS radical cation was determined according to Re et al. [53]. Absorbance at 734 nm was measured using a spectrophotometer (UV-1700, Shimadzu, Tokyo, Japan), and 100% of methanol was used as a control. The ABTS assay was calculated using the Equation (1).

### 3.11. Ferric Reducing/Antioxidant Power Assay (FRAP)

The FRAP assay was determined according to Benzie and Strain [54]. Absorbance at 593 nm was measured using a spectrophotometer (UV-1700, Shimadzu, Tokyo, Japan). The FRAP assay was expressed in milligrams of FeSO_4_ per gram of dry weight (mg FeSO_4_/g DW).

### 3.12. Inhibitory Activity against Enzyme α-Amylase

The method of Xiao et al. [55] was used to test for the inhibitory activity of the α-amylase, using potato starch as substrate. A microplate reader (Asys UVM 340, Biochrom, Cambridge, UK) was used at 650 nm. Acarbose was the control. The % inhibition of α-amylase was determined according to the following, Equation (1).

### 3.13. Inhibitory Activity against Enzyme α-Glucosidase

The α-glucosidase inhibition was determined according to Wang and Zhao [56]. We used a microplate reader (Asys UVM 340, Biochrom, Cambridge, UK) at 405 nm. The % inhibition of α-glucosidase was determined using Equation (1).

### 3.14. Evaluation of Anti-AGEs Formation Activity

Inhibitory capacities of AGEs formation were measured by the method of Vinson and Howard [57]. The extent of fluorescent AGEs formed was determined with a fluorescent spectrometer (F-7100, Hitachi, Tokyo, Japan): excitation wavelength, 330 nm; emission wavelength, 410 nm. Percent anti-AGEs formation was based on the resulting fluorescent intensity (FI) by this equation:

% Inhibition = [1 − (FIsample − FIsample blank)/(FIcontrol − FIcontrol blank)] × 100,(2)

where FIsample = fluorescent intensity of the sample, FIsample blank = fluorescent intensity of sample blank, FIcontrol = fluorescent intensity of control, and FIcontrol blank = fluorescent intensity of sample control blank.

### 3.15. Statistical Analysis

An SPSS program served to interpret results (IBM SPSS, Chicago, IL, USA), as the average of three replicates of standard deviation (SD). One-way analysis of variance (ANOVA) was used with the least significant difference (LSD) measure, with a significance level of *p* 0.05.

## 4. Conclusions

Our study has demonstrated that enzymatic extraction of silk cocoons gives the greater values of protein content, amino acids, total phenolic content (TPC), total flavonoid content (TFC), DPPH radical-scavenging activity, ABTS radical scavenging capacity assay, FRAP assay, anti-α-amylase, anti-α-glucosidase, and anti-glycation when compared with water extraction. The limitations of the extraction method selected in this study were that six hours of water extraction resulted in a longer time and more energy consumption. However, water extraction gave a more desirable taste if use as a functional drink is intended (less bitter, sweeter, and umami). In selecting the preferred extraction method, the enzyme-based procedure has a high potential for all aspects of the silk protein treatment, but there are disadvantages in that enzymes are expensive and that enzyme treatment produces a bitter taste. Alternatively, the enzyme treatment could be more suitable if used as concentrated nutraceuticals in other forms, such as encapsulated, cosmetics and nutraceuticals, rather than for direct consumption, e.g., as a drink.

## Figures and Tables

**Table 1:** The effect of extraction method on amino-acid and protein contents of SPE.

Parameters		Extraction Method		
		Control	Water	Enzyme
Amino acid content (µg/g DW) Essential amino acids	Phenylalanine	1.39 ± 0.06 ^c^	10.37 ± 0.17 ^b^	35.11 ± 0.12 ^a^
	Valine	2.04 ± 0.09 ^c^	5.31 ± 0.08 ^b^	30.60 ± 0.16 ^a^
	Tryptophan	1.73 ± 0.15 ^c^	3.34 ± 0.27 ^b^	28.28 ± 0.28 ^a^
	Threonine	1.44 ± 0.09 ^c^	24.41 ± 0.17 ^b^	32.21 ± 0.21 ^a^
	Isoleucine	0.88 ± 0.02 ^c^	2.86 ± 0.11 ^b^	18.80 ± 0.13 ^a^
	Methionine	0.53 ± 0.07 ^c^	2.72 ± 0.08 ^b^	6.96 ± 0.12 ^a^
	Histidine	0.17 ± 0.06 ^c^	10.60 ± 0.13 ^b^	15.57 ± 0.33 ^a^
	Arginine	0.60 ± 0.07 ^c^	5.39 ± 0.15 ^b^	43.47 ± 0.55 ^a^
	Lysine	1.39 ± 0.17 ^c^	3.42 ± 0.19 ^b^	21.16 ± 0.07 ^a^
	Leucine	0.46 ± 0.09 ^c^	2.53 ± 0.11 ^b^	12.67 ± 0.10 ^a^
Non-essential amino acids	Glycine	2.93 ± 0.06 ^c^	36.45 ± 0.34 ^b^	54.55 ± 0.36 ^a^
	Glutamic acid	0.92 ± 0.04 ^c^	22.66 ± 0.23 ^b^	30.68 ± 0.18 ^a^
	Aspartic acid	1.86 ± 0.07 ^c^	26.10 ± 0.18 ^b^	31.72 ± 0.25 ^a^
	Glutamine	1.90 ± 0.04 ^c^	22.28 ± 0.05 ^b^	39.70 ± 0.14 ^a^
	Serine	1.98 ± 0.06 ^c^	10.15 ± 0.11 ^b^	31.17 ± 0.16 ^a^
	Tyrosine	0.73 ± 0.05 ^c^	3.26 ± 0.19 ^b^	38.18 ± 0.12 ^a^
	Alanine	0.83 ± 0.05 ^c^	5.27 ± 0.08 ^b^	28.77 ± 0.18 ^a^
	Asparagine	1.14 ± 0.09 ^c^	5.81 ± 0.17 ^b^	16.98 ± 0.09 ^a^
Total amino acids		22.87 ± 0.11 ^c^	202.96 ± 0.21 ^b^	516.61 ± 0.32 ^a^
Protein content (mg/g)		0.84 ± 0.07 ^c^	2.15 ± 0.05 ^b^	3.26 ± 0.08 ^a^

Values are expressed as mean ± standard deviation (*n* = 3). Means with different letters in the row were significant differences at *p* < 0.05.

## Data Availability

All data is contained within the article.

## Sample Availability

Not available.
